# Decoding Thrombocytopenia in Dengue: Platelet Indices Define Mechanistic Phenotypes and Predict Bleeding Risk

**DOI:** 10.7759/cureus.110292

**Published:** 2026-06-05

**Authors:** Bashir A Bashir

**Affiliations:** 1 Department of Hematology, Port Sudan Ahlia University, Port Sudan, SDN

**Keywords:** bleeding risk, dengue, immature platelet fraction, platelet indices, thrombocytopenia phenotypes

## Abstract

Background: Thrombocytopenia is a characteristic of dengue infection; however, the underlying mechanisms of these phenotypes remain underexplored clinically. Platelet indices may serve as a swift and accessible method for mechanistic distinction and risk assessment.

Objective: The objective of this study was to evaluate platelet indices for mechanistic categorization of thrombocytopenia phenotypes in dengue patients, assess their association with bleeding risk, determine their diagnostic performance for bleeding prediction, and develop a preliminary Dengue Bleeding Risk Score (DBRS) for risk stratification.

Method: A case-control study included 334 dengue cases and 101 controls. We examined platelet count (PLT), plateletcrit (PCT), immature platelet fraction (IPF), mean platelet component (MPC), mean platelet volume (MPV), platelet distribution width (PDW), platelet large cell ratio (PLCR), platelet large cell count (PLCC), and coagulation parameters. Thrombocytopenia was classified into four phenotypes: hyperdestructive, inadequate response, hypoproductive, and lazy-suppressed, based on PLT and IPF. Platelet indices, coagulation parameters, and clinical outcomes were analyzed. Logistic regression and receiver operating characteristic (ROC) curve analyses were performed to evaluate bleeding risk.

Results: Thrombocytopenia was present in 82.9% of patients, with inadequate response being the most common phenotype (55.7%). A significant shift toward the hyperdestructive phenotype was observed as disease severity increased (p < 0.001). Bleeding differed significantly across phenotypes, with the highest frequency in the hyperdestructive group (57.1%). In unadjusted analysis, this phenotype was strongly associated with bleeding (OR 13.25, 95% CI 4.11-42.71, p < 0.001); however, this association was not independent after adjustment for disease severity (OR 2.83, 95% CI 0.59-13.60, p = 0.194). Severe dengue remained the strongest predictor of bleeding (adjusted OR 41.83, 95% CI 14.41-121.46, p < 0.001). IPF and MPC demonstrated moderate performance in predicting bleeding (area under the curve (AUC) 0.723 and 0.718, respectively).

Conclusion: Thrombocytopenia in dengue is a heterogeneous, mechanism-based condition characterized by diverse phenotypes; however, bleeding risk is primarily determined by disease severity rather than phenotype alone. The incorporation of platelet indices into a composite model, such as the DBRS, may provide a more comprehensive approach to risk stratification than platelet count alone, although further validation in independent cohorts is required.

## Introduction

Dengue poses a major public health challenge in tropical and subtropical regions, with incidence rising and its geographic range expanding. This effect is especially strong in resource-poor settings, such as coastal areas like the Red Sea State, where frequent epidemics strain healthcare systems [1]. Thrombocytopenia is a key feature of dengue infection and worsens both illness severity and bleeding risk. This condition arises from both peripheral destruction and reduced platelet production [1,2].

Despite its clinical significance, the standard assessment of thrombocytopenia in dengue focuses on platelet count. This quantitative method alone does not adequately capture the underlying pathophysiological mechanisms. These mechanisms could impact disease progression and hemorrhagic risk. Recent evidence shows that platelet indices, especially the immature platelet fraction (IPF), indicate bone marrow activity and platelet turnover. These indices provide insight into the processes underlying thrombocytopenia [3-5]. However, the use of these measures for dengue remains limited, particularly in dengue-endemic regions of Africa.

In places like the Red Sea State, where advanced diagnostics are scarce, clinicians urgently need simple, reliable, and accessible biomarkers for better decision-making. Distinguishing hyperdestructive, inadequate response, hypoproductive, and lazy-suppressed thrombocytopenia phenotypes helps identify patients at higher bleeding risk and guide therapy.

We expected platelet indices to distinguish thrombocytopenia subtypes in dengue and predict clinical outcomes, especially bleeding. This study primarily aimed to evaluate platelet indices for the mechanistic classification of thrombocytopenia phenotypes in dengue patients and to assess their association with bleeding manifestations. Secondary objectives were to determine the diagnostic performance of platelet indices for bleeding prediction and to develop a preliminary Dengue Bleeding Risk Score (DBRS) for clinical risk stratification.

## Materials and methods

Study design and setting

This hospital-based analytical case-control study at Port Sudan Teaching Hospital highlights the importance of rigorous research design and the expertise of healthcare professionals involved. This is a tertiary healthcare setting in Sudan, including patients from dengue-endemic regions such as the Red Sea State, to evaluate the role of platelet indices in differentiating thrombocytopenia mechanisms in dengue patients. The trial spanned 14 months, from November 2024 to December 2025.

Study population

The study comprised 435 participants: 334 patients with confirmed dengue infection (cases), defined by positive diagnostic tests for non-structural protein-1 antigen (NS1) (Finecare™, Lot F28215116A0; Guangzhou Wondfo Biotech Co., Ltd., Guangzhou, China), and 101 healthy individuals (controls). Cases were characterized by acute febrile illness with symptoms like headache, myalgia, arthralgia, or hemorrhagic signs, confirmed through diagnostic testing to ensure accuracy. The use of NS1 testing for diagnosis demonstrates the study's commitment to accurate testing.

Controls were selected from the same geographic region. They were healthy, had not had a fever recently, and showed no clinical signs of dengue infection. Cases and controls were frequency-matched by age group and sex to ensure comparability between groups. The controls were younger and more homogeneous, potentially introducing residual confounding despite the corrections implemented.

Dengue patients were clinically categorized into dengue fever (DF), dengue hemorrhagic fever (DHF), and dengue shock syndrome (DSS) [1], facilitating evaluation throughout the entire spectrum of disease severity. This classification is crucial, as it illustrates the progressive pathophysiological alterations associated with dengue infection and facilitates the linkage between laboratory results and clinical outcomes.

Inclusion and Exclusion Criteria

Patients of all ages and both genders with confirmed dengue infections were included. Controls were clinically healthy and had no indications of dengue infection. Participants were excluded if they had concurrent infections, including malaria, typhoid, or viral hepatitis, established chronic liver disease, pre-existing coagulation disorders, or a history of anticoagulant treatment or recent blood transfusion before sample collection.

Bleeding evaluation

All individuals underwent a comprehensive clinical evaluation. We documented demographic data, encompassing age and gender, and conducted a comprehensive clinical assessment. Extreme care was devoted to identifying indicators of hemorrhage, such as epistaxis, gingival hemorrhage, hematuria, gum bleeding, and gastrointestinal bleeding.

Data collection

Clinical and analytical data were thoroughly gathered to facilitate a thorough assessment of each patient. Demographic characteristics, such as age and sex, were documented to evaluate baseline comparability and potential risk factors. Moreover, essential clinical characteristics, including fever, headache, myalgia, rash, and hemorrhagic signs, were recorded, as these symptoms constitute the fundamental clinical presentation of dengue and are strongly associated with illness severity and sequelae. The incorporation of hemorrhagic signs was particularly significant, considering their importance in delineating severe dengue and their strong correlation with thrombocytopenia and platelet dysfunction.

Laboratory analysis

Venous blood specimens were obtained from all subjects under aseptic circumstances. Approximately 2-3 mL of peripheral venous blood was collected via normal phlebotomy techniques and transferred into tri-potassium ethylenediaminetetraacetate (K₃EDTA)-anticoagulated tubes, which are advised for complete blood count (CBC) analysis to maintain platelet count and morphology while preventing clot formation. Samples were examined within the prescribed time limit to reduce pre-analytical variability.

In CBC, platelet indices were assessed with a fully automated 6-part hematology analyzer (Dymind DH-800 Auto Hematology Line; Shenzhen Dymind Biotechnology Co., Ltd., Guangdong, China), which provides comprehensive platelet parameters, including platelet count (PLT), mean platelet component (MPC), mean platelet volume (MPV), immature platelet fraction (IPF), platelet distribution width (PDW), PDW-standard deviation (PDWsd), plateletcrit (PCT), platelet large cell ratio (PLCR), and platelet large cell count (PLCC).

The analyzer operates on advanced principles that combine impedance and optical fluorescence technologies, allowing for accurate differentiation of blood cell groups and reliable evaluation of platelet indices. Internal quality control procedures were systematically executed in compliance with manufacturer guidelines to ensure analytical precision and result reproducibility.

We also assessed other coagulation markers, including prothrombin time (PT), partial thromboplastin time (PTT), and fibrinogen concentration. PT and PTT were assessed utilizing Hart’s Reagent. At the same time, fibrinogen was evaluated using the FIBROSCREEN™ thrombin time test (Tulip Diagnostics Pvt. Ltd., Goa, India), all of which were conducted on a URIT-610 Coagulation Analyzer (URIT Medical Electronic Co. Ltd., Guilin, Guangxi, China).

Definition of thrombocytopenia phenotypes

Thrombocytopenia was characterized by a platelet count below 150×10⁹/L, in accordance with established hematological standards. Phenotypes were assigned based on PLT and IPF-based marrow response. Hyperdestructive thrombocytopenia was defined as low PLT with elevated IPF, indicating increased peripheral platelet destruction with compensatory thrombopoiesis. Conversely, hypoproductive thrombocytopenia was defined as low PLT with low IPF, indicating reduced platelet production. The inadequate response phenotype was characterized by a suboptimal marrow response relative to the degree of thrombocytopenia, whereas the lazy-suppressed phenotype was characterized by a minimal or absent marrow response despite thrombocytopenia.

**Figure 1:**
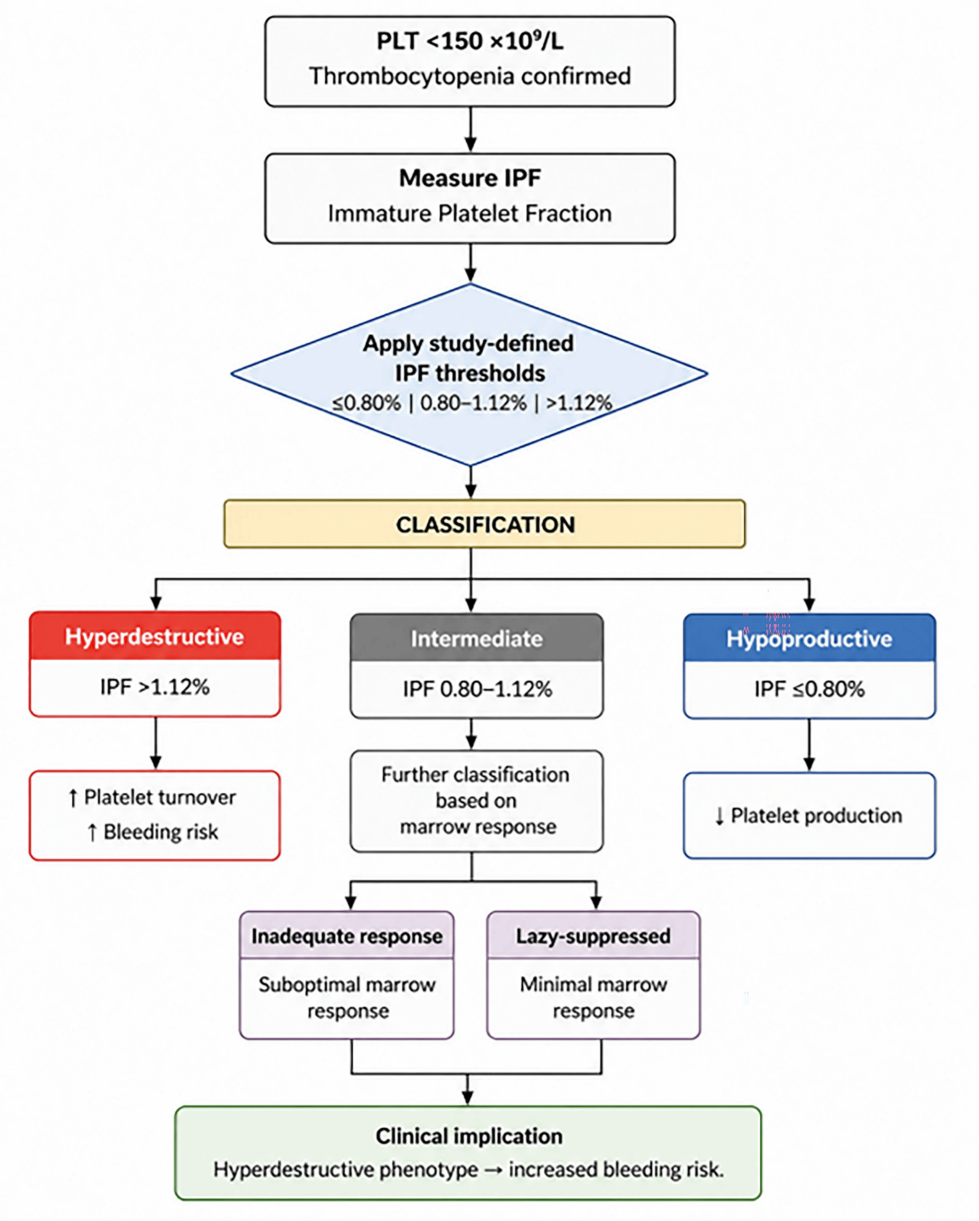
Study-derived classification of thrombocytopenia phenotypes in dengue based on IPF Patients with platelet count <150 ×10⁹/L were stratified into hyperdestructive, intermediate, and hypoproductive patterns based on IPF thresholds (≤0.80%, 0.80–1.12%, >1.12%), with further subclassification of the intermediate group into inadequate response and lazy-suppressed phenotypes. The hyperdestructive phenotype was associated with increased bleeding risk. IPF, immature platelet fraction; PLT, platelet count Figure created by the authors using Microsoft PowerPoint (Microsoft Corporation, Redmond, Washington, United States).

This mechanistic classification framework was designed to capture the full spectrum of thrombocytopenia mechanisms in dengue and to improve mechanistic interpretation beyond conventional binary models. A stepwise diagnostic approach based on PLT and IPF thresholds was applied. The IPF thresholds (≤0.80%, 0.80-1.12%, and >1.12%) were study-derived, data-driven cutoffs based on the distribution of IPF values within the study cohort and were used to distinguish distinct platelet kinetic patterns. These thresholds were selected to reflect biologically plausible differences in platelet production and peripheral consumption and were applied consistently throughout the analysis. As this classification framework was developed from a single study cohort, the thresholds should be considered exploratory and require validation in independent populations before broader clinical application. The classification process is illustrated in Figure 1.

Data analysis

Data were analyzed using IBM SPSS Statistics for Windows, version 27.0 (IBM Corp., Armonk, New York, United States). To ensure reliability, all continuous variables were carefully assessed for normality using both the Shapiro-Wilk and Kolmogorov-Smirnov (K-S) tests, complemented by visual inspection of histograms and Q-Q plots. The results of normality testing demonstrated that most hematological and platelet indices, including PLT, PCT, IPF, MPC, PDW, PLCR, and PLCC, were not normally distributed (p < 0.05 for both the Shapiro-Wilk and K-S tests). Consequently, these variables were reported as medians and interquartile ranges (IQRs). In contrast, variables that approximated a normal distribution were described using mean ± standard deviation where appropriate.

For comparisons between two independent groups (e.g., dengue vs control or thrombocytopenia phenotypes), the Mann-Whitney U test and Kruskal-Wallis test were employed for continuous variables, reflecting the non-normal data distribution, while categorical variables were analyzed with the Chi-square or Fisher’s exact test when expected counts were small. For more than two groups (DF, DHF, DSS), the Kruskal-Wallis test was used, followed by post hoc pairwise comparisons to ensure accurate interpretation.

Correlation between continuous variables was assessed using Spearman’s rank correlation coefficient (ρ) due to the non-parametric nature of the data. To identify predictors of bleeding, logistic regression analysis was performed, and results were expressed as odds ratios (ORs) with 95% confidence intervals (CIs). Age was examined as a continuous variable within a logistic regression model. Model stability and clinical plausibility were assessed during logistic regression analysis. Due to the small number of DSS cases and complete separation, disease severity was modeled as a binary variable (DF versus severe dengue (DHF/DSS)) in the adjusted analysis. The diagnostic performance of platelet indices, particularly IPF, was evaluated using receiver operating characteristic (ROC) curve analysis, with calculation of the area under the curve (AUC), determination of optimal cutoff values based on the Youden index, and assessment of sensitivity and specificity.

The DBRS was developed as an exploratory risk-stratification model and was not subjected to formal internal or external validation; therefore, its performance should be interpreted cautiously pending independent validation. The weighting of individual DBRS components was based on the relative strength of their observed associations with bleeding in the present dataset, incorporating findings from logistic regression analyses, ROC performance, and biological plausibility. When overall group comparisons were significant, post-hoc pairwise comparisons were performed to facilitate interpretation. The dataset was reviewed for completeness before analysis, and no variables included in the final analyses contained missing data requiring imputation. A two-tailed p-value < 0.05 was set as statistically significant throughout the analysis.

Ethical consideration

This study was approved in accordance with the ethical principles set forth in the Declaration of Helsinki and its subsequent revisions. The Research Ethics Committee of Port Sudan Ahlia University granted ethical approval (REC-PAU-18/07, dated September 15, 2024). Informed written consent was secured from all participants or their legal guardians prior to registration. Participant confidentiality and data privacy were rigorously upheld during the study. 

## Results

Demographics and clinical characteristics

A total of 435 participants were included in the present study, comprising 334 dengue patients (cases) and 101 controls. Among the 334 cases, the diagnostic spectrum was predominantly DF, accounting for 289 patients (86.5%), followed by DHF in 43 patients (12.9%) and DSS in two patients (0.6%), indicating that the cohort was largely composed of uncomplicated dengue with a smaller proportion of severe clinical phenotypes.

The study population was relatively young; however, a statistically significant difference in age distribution was observed between the two groups. Dengue patients had a median age of 27.0 years (IQR 19.0-40.0) compared with 22.0 years (IQR 20.0-23.0) in controls (Mann-Whitney U test, p < 0.001). Despite this difference, both groups remained predominantly young adults. Sex distribution was comparable between cases and controls, with a slight male predominance in both groups. Among dengue patients, 218 (65.3%) were male, and 116 (34.7%) were female, whereas among controls, 64 (63.4%) were male and 37 (36.6%) were female. This difference was not statistically significant (Chi-square test, p = 0.816), confirming adequate comparability.

Clinically, dengue patients presented with fever in all cases (n=334, 100%), whereas none of the controls (0/101) did, showing a highly significant difference (p < 0.001). Headaches were reported in 282 cases (84.4%) versus none in controls (p < 0.001), while joint pain occurred in 262 cases (78.4%) compared with none in controls (p < 0.001). Myalgia was observed in 156 cases (46.7%), again absent in controls (p < 0.001), and backache in 198 cases (59.3%) (p < 0.001). Retro-orbital pain was present in 69 cases (20.7%) (p < 0.001) and rash in 28 cases (8.4%) (p = 0.005) (Table 1).

**Table 1:** Demographic and clinical characteristics of study participants p < 0.05 indicates statistically significant differences. IQR, interquartile range

Variable	Dengue Cases (n = 334)	Controls (n = 101)	Odds Ratio/ (95% CI)	p-value
Age (years), median (IQR)	27.0 (19.0–40.0)	22.0 (20.0–23.0)	1.06 (1.03–1.08)	<0.001
Sex, n (%)			1.09 (0.68–1.73)	0.816
Male	218 (65.3%)	64 (63.4%)	—	—
Female	116 (34.7%)	37 (36.6%)	—	
Clinical Features, n (%)				
Fever	334 (100%)	0 (0%)	1.36×10⁵ (8.3×10³ – 2.2×10⁶)	<0.001
Headache	282 (84.4%)	0 (0%)	1.09×10³ (6.6×10¹ – 1.8×10⁴)	<0.001
Joint pain	262 (78.4%)	0 (0%)	735 (45.0 – 1.2×10⁴)	<0.001
Myalgia	156 (46.7%)	0 (0%)	178 (11.1 – 2.9×10³)	<0.001
Backache	198 (59.3%)	0 (0%)	295 (18.5 – 4.7×10³)	<0.001
Retro-orbital pain	69 (20.7%)	0 (0%)	53 (3.3 – 851)	<0.001
Rash	28 (8.4%)	0 (0%)	19 (1.2 – 302)	0.005

Hemorrhagic manifestations were observed in 34 of the 334 dengue patients (10.2%), while the majority (n=300, 89.8%) did not exhibit bleeding. The occurrence of bleeding differed significantly across diagnostic categories (chi-square test, p < 0.001). Bleeding was observed in nine of the 289 DF patients (3.1%), compared with 23 of the 43 DHF patients (53.5%) and both of the two DSS patients (100%), demonstrating a marked increase in hemorrhagic manifestations with disease severity. Despite this strong association, bleeding was not universally present even among severe cases, as nearly half of DHF patients (46.5%) did not exhibit hemorrhagic manifestations.

Platelet indices according to the diagnosis and clinical correlation

Before mechanistic classification, platelet indices were analyzed across diagnostic categories (DF, DHF, DSS) to evaluate their distribution along the clinical severity spectrum. Significant differences were observed in several platelet parameters (Table 2). PLT decreased progressively with disease severity, with a median of 94.0 ×10⁹/L (IQR 64.0-136.0) in DF, 46.0 ×10⁹/L (IQR 17.0-90.5) in DHF, and 18.0 ×10⁹/L (IQR 10.5-25.5) in DSS (Kruskal-Wallis p < 0.001). PCT showed a parallel decline, falling from 9.46 (IQR 6.37-13.92) in DF to 4.92 (IQR 1.78-8.98) in DHF and 1.78 (IQR 1.02-2.54) in DSS (p < 0.001), indicating progressive reduction in circulating platelet mass with increasing disease severity. Similarly, the IPF increased markedly with severity, rising from 1.06% (IQR 0.74-1.56) in DF to 2.17% (IQR 1.11-5.88) in DHF and reaching 18.18% (IQR 10.61-25.76) in DSS (p < 0.001), indicating a strong severity-dependent increase in platelet turnover.

**Table 2:** Platelet indices according to the dengue diagnosis *p-values calculated using the Kruskal–Wallis test for comparison across three groups (DF, DHF, DSS). Values are presented as median (interquartile range, IQR). p < 0.05 indicates statistically significant differences. DF, dengue fever; DHF, dengue hemorrhagic fever; DSS, dengue shock syndrome; PLT, platelet count; PCT, plateletcrit; IPF, immature platelet fraction; MPC, mean platelet component; MPV, mean platelet volume; PDW, platelet distribution width; PDWsd, platelet distribution width standard deviation; PLCC, platelet large cell count; PLCR, platelet large cell ratio; PT, prothrombin time; aPTT, activated partial thromboplastin time.

Parameter	DF (n = 289), median (IQR)	DHF (n = 43), median (IQR)	DSS (n = 2), median (IQR)	p-value*
PLT (×10⁹/L)	94.0 (64.0–136.0)	46.0 (17.0–90.5)	18.0 (10.5–25.5)	<0.001
PCT (%)	9.46 (6.37–13.92)	4.92 (1.78–8.98)	1.78 (1.02–2.54)	<0.001
IPF (%)	1.06 (0.74–1.56)	2.17 (1.11–5.88)	18.18 (10.61–25.76)	<0.001
MPC (g/dl)	0.11 (0.07–0.17)	0.26 (0.11–0.59)	1.60 (0.95–2.25)	<0.001
PLCC (×10⁹/L)	26.24 (16.10–37.82)	13.40 (5.51–24.07)	2.29 (1.61–2.96)	<0.001
MPV (fL)	10.2 (9.3–11.1)	9.9 (9.3–11.0)	9.35 (9.02–9.68)	0.517
PDW (%)	14.2 (12.3–17.7)	14.7 (12.3–18.3)	11.9 (10.4–13.5)	0.514
PDWsd (fL)	1.50 (1.18–1.83)	1.55 (1.27–1.87)	1.09 (0.99–1.20)	0.290
PLCR (%)	29.4 (23.2–36.9)	29.7 (24.3–37.7)	21.2 (16.1–26.3)	0.493

PLCC also demonstrated a significant decline across diagnostic groups, with median values of 26.24 (IQR 16.10-37.82) in DF, 13.40 (IQR 5.51-24.07) in DHF, and 2.29 (IQR 1.61-2.96) in DSS (p < 0.001). MPC showed a significant increase in severity, from 0.11 (IQR 0.07-0.17) in DF to 0.26 (IQR 0.11-0.59) in DHF and 1.60 (IQR 0.95-2.25) in DSS (p < 0.001). In contrast, no statistically significant differences were observed for MPV, which remained relatively stable across DF (10.2 fL (9.3-11.1)), DHF (9.9 fL (9.3-11.0)), and DSS (9.35 fL (9.02-9.68)) (p = 0.517). Similarly, PDW, PDWsd, and PLCR did not differ significantly between diagnostic groups (all p > 0.05), despite minor differences in distribution. 

**Figure 2:**
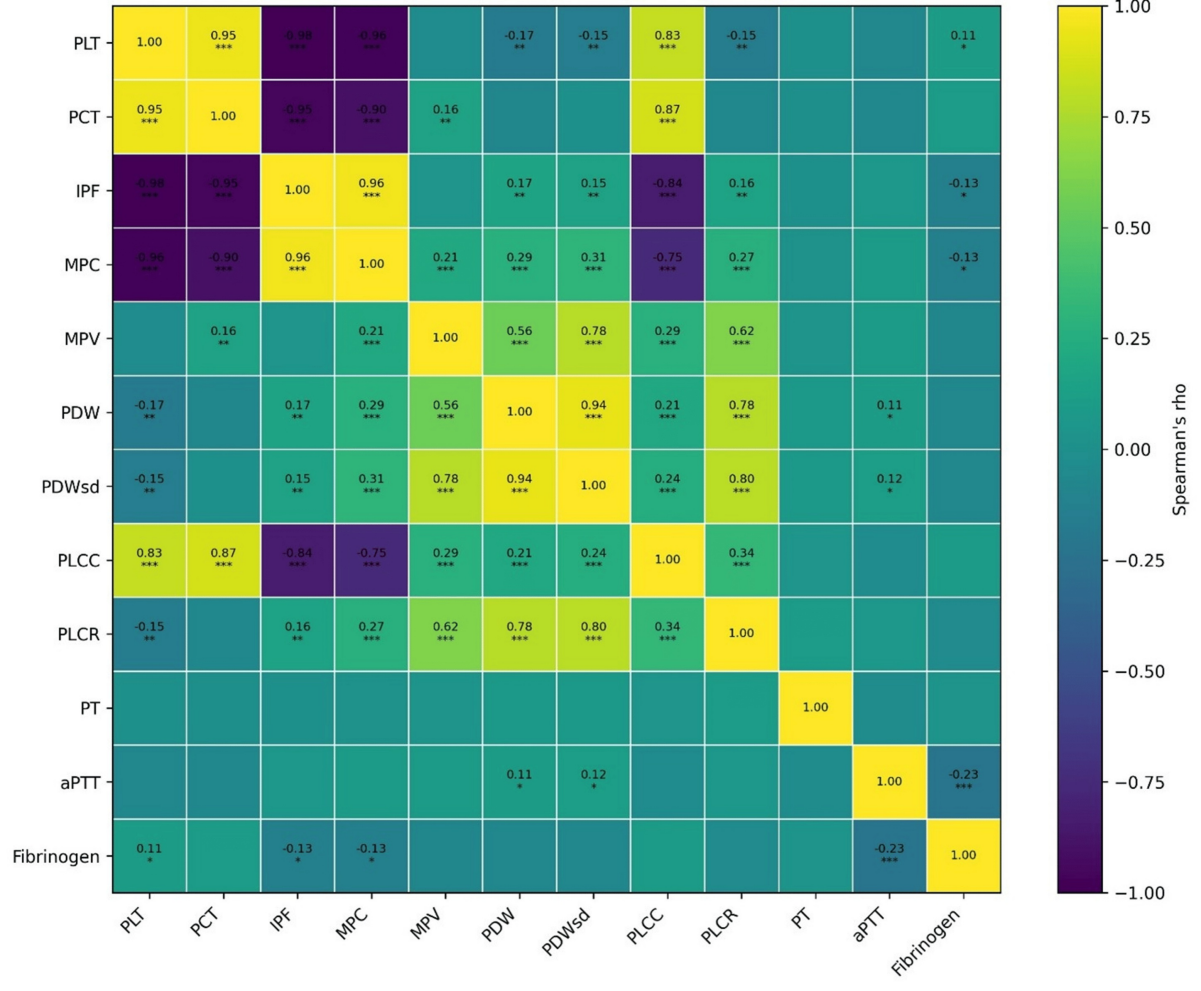
Correlation heatmap of platelet indices and coagulation parameters in dengue patients. The heatmap illustrates the correlation matrix between platelet indices and coagulation parameters. Strong positive and negative correlations highlight relationships between platelet production, turnover, and hemostatic function. PLT, platelet count; PCT, plateletcrit; IPF, immature platelet fraction; MPC, mean platelet component; MPV, mean platelet volume; PDW, platelet distribution width; PDWsd, platelet distribution width standard deviation; PLCC, platelet large cell count; PLCR, platelet large cell ratio; PT, prothrombin time; aPTT, activated partial thromboplastin time

An inverse association between IPF and PLT was observed, suggesting heightened platelet turnover in hyperdestructive thrombocytopenia. PCT exhibited a robust positive connection with PLT, indicating the overall circulating platelet mass (Figure 2).

Platelet indices and thrombocytopenia classification

Thrombocytopenia was present in a significant proportion of the cases (n=277, 82.9%) and was subsequently categorized mechanistically based on PLT and IPF. Among the classification models, patients were classified into four phenotypes: inadequate response (n=186, 55.7%), hyperdestructive (n=14, 4.2%), hypoproductive (n=81, 24.3%), and lazy-suppressed thrombocytopenia (n=53, 15.9%).

Phenotype distribution across disease severity

A clear shift in the distribution of thrombocytopenia phenotypes was observed across diagnostic categories. In the 289 cases with DF, the inadequate response phenotype predominated (n=162, 56.1%), followed by hypoproductive (n=73, 25.3%) and lazy-suppressed (n=51, 17.6%), while hyperdestructive was rare (n=3, 1.0%). In the 43 cases with DHF, the hyperdestructive phenotype increased markedly (n=10, 23.3%), alongside the inadequate response phenotype (n=23, 53.5%), while hypoproductive (18.6%) and lazy-suppressed (4.7%) phenotypes were less frequent. In DSS, although limited by a small sample size (n = 2), both hyperdestructive and inadequate response phenotypes accounted for 50% each.

Overall, phenotype distribution differed significantly across severity groups (Pearson χ² = 59.684, df = 6, p < 0.001). However, 41.7% of cells had expected counts < 5, indicating violation of chi-square assumptions. Therefore, results were interpreted cautiously, and Fisher’s exact test confirmed a significant difference between DF and DHF (p = 0.015).

Platelet indices according to thrombocytopenia phenotypes

Quantitative comparison of platelet indices demonstrated marked differences across thrombocytopenia phenotypes (Table 3). Phenotype classification was performed using the predefined algorithm (Figure 1).

**Table 3:** Platelet indices and coagulation parameters across thrombocytopenia phenotypes. Bold values indicate p-values calculated using the Kruskal–Wallis test (p < 0.05). PLT, platelet count; PCT, plateletcrit; IPF, immature platelet fraction; MPC, mean platelet component; MPV, mean platelet volume; PDW, platelet distribution width; PDWsd, platelet distribution width standard deviation; PLCC, platelet large cell count; PLCR, platelet large cell ratio; PT, prothrombin time; aPTT, activated partial thromboplastin time; IQR, interquartile range.

Parameter	Inadequate (n=186), median (IQR)	Hyperdestructive (n=14), median (IQR)	Hypoproductive (n=81), median (IQR)	Lazy-suppressed (n=53), median (IQR)	H-value	p-value
PLT (×10⁹/L)	66.5 (41.0–83.8)	11.0 (9.0–13.8)	120.0 (109.0–130.0)	167.0 (156.0–192.0)	72.3	<0.001
PCT (%)	6.83 (4.18–8.61)	1.15 (0.85–1.36)	12.31 (10.71–14.06)	17.26 (14.99–19.69)	70.1	<0.001
IPF (%)	1.50 (1.19–2.44)	9.09 (7.28–11.11)	0.83 (0.76–0.92)	0.60 (0.52–0.64)	95.4	<0.001
MPC (g/dL)	0.16 (0.12–0.24)	0.93 (0.79–0.99)	0.087 (0.077–0.096)	0.058 (0.052–0.062)	88.2	<0.001
MPV (fL)	10.4 (9.3–11.0)	10.05 (8.9–10.9)	10.1 (9.2–11.4)	10.0 (9.4–10.6)	2.1	0.548
PDW (%)	14.7 (12.4–17.7)	17.0 (14.3–19.8)	13.9 (12.1–17.2)	13.3 (12.1–15.3)	7.9	0.048
PDWsd (fL)	1.56 (1.20–1.87)	1.71 (1.39–2.09)	1.39 (1.18–1.81)	1.31 (1.17–1.65)	6.5	0.070
PLCC (×10⁹/L)	31.35 (23.63–37.58)	34.0 (27.8–38.0)	28.0 (20.9–35.6)	27.4 (23.2–33.2)	3.8	0.093
PLCR (%)	17.7 (12.52–25.48)	3.70 (2.95–4.26)	31.0 (26.21–43.47)	49.6 (38.10–65.03)	15.6	<0.001
PT (s)	13.65 (12.5–15.8)	14.75 (14.0–16.08)	13.9 (12.5–15.1)	14.0 (12.2–15.9)	2.8	0.424
aPTT (s)	32.0 (26.9–38.8)	34.5 (28.0–41.0)	31.0 (27.0–36.0)	31.5 (27.0–37.5)	1.9	0.661
Fibrinogen (mg/dL)	264 (188–359)	250 (170–350)	270 (200–380)	260 (190–360)	3.1	0.288

PLT differed significantly between groups (p < 0.001), with the lowest median value observed in the hyperdestructive phenotype at 11.0 ×10⁹/L (IQR 9.0-13.8; 95% CI 9.0-14.0), followed by the inadequate response phenotype at 66.5 ×10⁹/L (IQR 41.0-83.8; 95% CI 58.5-71.0), the hypoproductive phenotype at 120.0 ×10⁹/L (IQR 109.0-130.0; 95% CI 113.0-125.0), and the lazy-suppressed phenotype at 167.0 ×10⁹/L (IQR 156.0-192.0; 95% CI 163.0-179.0). PCT showed the same pattern (p < 0.001), with medians of 1.15 (IQR 0.85-1.36; 95% CI 0.89-1.35) in the hyperdestructive group, 6.83 (IQR 4.18-8.61; 95% CI 6.05-7.45) in the inadequate response group, 12.31 (IQR 10.71-14.06; 95% CI 11.48-12.90) in the hypoproductive group, and 17.26 (IQR 14.99-19.69; 95% CI 16.14-18.20) in the lazy-suppressed group.

IPF was the strongest discriminator among thrombocytopenia phenotypes (p < 0.001). The hyperdestructive group had the highest median IPF at 9.09% (IQR 7.28-11.11; 95% CI 7.14-11.11), followed by the inadequate response group at 1.50% (IQR 1.19-2.44; 95% CI 1.41-1.71), the hypoproductive group at 0.83% (IQR 0.76-0.92; 95% CI 0.80-0.87), and the lazy-suppressed group at 0.60% (IQR 0.52-0.64; 95% CI 0.56-0.61). MPC also differed significantly across phenotypes (p < 0.001), with values highest in hyperdestructive thrombocytopenia (0.93 (IQR 0.79-0.99; 95% CI 0.79-0.99)), followed by inadequate response (0.16 (IQR 0.12-0.24; 95% CI 0.15-0.18)), hypoproductive (0.087 (IQR, 0.077-0.096; 95% CI 0.082-0.089)), and lazy-suppressed thrombocytopenia (0.058 (IQR, 0.052-0.062; 95% CI 0.054-0.061)).

Among platelet size and heterogeneity indices, MPV did not differ significantly between groups (p = 0.548), with median values of 10.4 fL (IQR 9.3-11.0; 95% CI 10.0-10.7) in inadequate response, 10.05 fL (IQR 8.9-10.9; 95% CI 8.9-11.0) in hyperdestructive, 10.1 fL (IQR 9.2-11.4; 95% CI 9.8-10.5) in hypoproductive, and 10.0 fL (IQR 9.4-10.6; 95% CI 9.7-10.3) in lazy-suppressed thrombocytopenia. In contrast, PDW differed significantly across phenotypes (p = 0.048) and was highest in the hyperdestructive group at 17.0 (IQR 14.3-19.8; 95% CI 14.1-20.0), compared with 14.7 (IQR 12.4-17.7; 95% CI 14.1-15.3) in inadequate response, 13.9 (IQR 12.1-17.2; 95% CI 12.9-14.8) in hypoproductive, and 13.3 (IQR 12.1-15.3; 95% CI 12.5-14.7) in lazy-suppressed thrombocytopenia. PDWsd showed a similar trend but did not reach statistical significance (p = 0.070), with medians of 1.71 (IQR 1.39-2.09; 95% CI 1.38-2.06), 1.56 (IQR 1.20-1.87; 95% CI 1.47-1.63), 1.39 (IQR 1.18-1.81; 95% CI 1.31-1.56), and 1.31 (IQR 1.17-1.65; 95% CI 1.25-1.50) in hyperdestructive, inadequate response, hypoproductive, and lazy-suppressed groups, respectively.

PLCR showed a borderline difference across phenotypes (p = 0.093), with median values of 34.0% (IQR 27.8-38.0; 95% CI 28.2-38.0) in the hyperdestructive group, 31.35% (IQR 23.63-37.58; 95% CI 27.9-32.7) in inadequate response, 28.0% (IQR 20.9-35.6; 95% CI 24.8-29.7) in hypoproductive, and 27.4% (IQR 23.2-33.2; 95% CI 24.9-31.4) in lazy-suppressed thrombocytopenia. PLCC differed significantly between phenotypes (p < 0.001), with the lowest value in hyperdestructive thrombocytopenia at 3.70 ×10⁹/L (IQR 2.95-4.26; 95% CI 2.94-4.32), followed by inadequate response at 17.7 ×10⁹/L (IQR 12.52-25.48; 95% CI 15.83-19.22), hypoproductive at 31.0 ×10⁹/L (IQR 26.21-43.47; 95% CI 29.0-35.53), and the highest value in the lazy-suppressed phenotype at 49.6 ×10⁹/L (IQR 38.10-65.03; 95% CI 43.09-56.10).

Coagulation parameters did not differ significantly across phenotypes. PT values were 13.65 s (IQR 12.5-15.8; 95% CI 13.35-14.0) in the inadequate response group, 14.75 s (IQR 14.0-16.08; 95% CI 14.0-16.1) in the hyperdestructive group, 13.9 s (IQR 12.5-15.1; 95% CI 13.20-14.5) in the hypoproductive group, and 14.0 s (IQR 12.2-15.9; 95% CI 12.6-15.2) in the lazy-suppressed group (p = 0.424). Similarly, aPTT (p = 0.661) and fibrinogen (p = 0.288) showed no statistically significant variation between groups.

Association between thrombocytopenia phenotypes and bleeding

Bleeding differed significantly across thrombocytopenia phenotypes (p < 0.001). The highest frequency was observed in the hyperdestructive phenotype, affecting 8/14 patients (57.1%), compared with 17/186 (9.1%) in the inadequate response group, 4/81 (4.9%) in the hypoproductive group, and 5/53 (9.4%) in the lazy-suppressed phenotype (Table 4).

**Table 4:** Platelet indices and coagulation parameters according to thrombocytopenia phenotypes †Chi-square test (χ²), *Kruskal–Wallis test (H) (p < 0.05). Although bleeding frequency differed significantly across thrombocytopenia phenotypes (p < 0.001), multivariable logistic regression analysis demonstrated that this association was not independent after adjustment for disease severity, which remained the strongest predictor of bleeding. PLT, platelet count; PCT, plateletcrit; IPF, immature platelet fraction; MPC, mean platelet component; MPV, mean platelet volume; PDW, platelet distribution width; PDWsd, platelet distribution width standard deviation; PLCC, platelet large cell count; PLCR, platelet large cell ratio; PT, prothrombin time; aPTT, activated partial thromboplastin time; IQR, interquartile range.

Parameter	Hyperdestructive (n=14), median (IQR)	Inadequate Response (n=186), median (IQR)	Hypoproductive (n=81), median (IQR)	Lazy-Suppressed (n=53), median (IQR)	H / χ²	p-value*
Bleeding, n (%)	8 (57.1%)	17 (9.1%)	4 (4.9%)	5 (9.4%)	59.68	<0.001^†^
PLT (×10⁹/L)	11.0 (9.0–13.8)	66.5 (41.0–83.8)	120.0 (109.0–130.0)	167.0 (156.0–192.0)	72.3	<0.001
PCT (%)	1.15 (0.85–1.36)	6.83 (4.18–8.61)	12.31 (10.71–14.06)	17.26 (14.99–19.69)	70.1	<0.001
IPF (%)	9.09 (7.28–11.11)	1.50 (1.19–2.44)	0.83 (0.76–0.92)	0.60 (0.52–0.64)	95.4	<0.001
MPC (g/dl)	0.93 (0.79–0.99)	0.16 (0.12–0.24)	0.087 (0.077–0.096)	0.058 (0.052–0.062)	88.2	<0.001
MPV (fL)	10.05 (8.9–10.9)	10.4 (9.3–11.0)	10.1 (9.2–11.4)	10.0 (9.4–10.6)	2.1	0.548
PDW (%)	17.0 (14.3–19.8)	14.7 (12.4–17.7)	13.9 (12.1–17.2)	13.3 (12.1–15.3)	7.9	0.048
PDWsd (fL)	1.71 (1.39–2.09)	1.56 (1.20–1.87)	1.39 (1.18–1.81)	1.31 (1.17–1.65)	6.5	0.070
PLCC (×10⁹/L)	34.0 (27.8–38.0)	31.35 (23.63–37.58)	28.0 (20.9–35.6)	27.4 (23.2–33.2)	3.8	0.093
PLCR (%)	3.70 (2.95–4.26)	17.7 (12.52–25.48)	31.0 (26.21–43.47)	49.6 (38.10–65.03)	15.6	<0.001
PT (s)	14.75 (14.0–16.08)	13.65 (12.5–15.8)	13.9 (12.5–15.1)	14.0 (12.2–15.9)	2.8	0.424
aPTT (s)	34.5 (28.0–41.0)	32.0 (26.9–38.8)	31.0 (27.0–36.0)	31.5 (27.0–37.5)	1.9	0.661
Fibrinogen (mg/dL)	250 (170–350)	264 (188–359)	270 (200–380)	260 (190–360)	3.1	0.288

In unadjusted binary logistic regression analysis, using the inadequate response phenotype as the reference category, the hyperdestructive phenotype was strongly associated with bleeding (OR 13.25, 95% CI 4.11-42.71, p < 0.001). In contrast, the hypoproductive phenotype (OR 0.52, 95% CI 0.17-1.59, p = 0.248) and the lazy-suppressed phenotype (OR 1.04, 95% CI 0.36-2.95, p = 0.948) were not significantly associated with bleeding.

To account for potential confounding, a multivariable logistic regression model was constructed, including age, sex, platelet count, and disease severity. Due to the small number of DSS cases and complete separation, disease severity was modeled as a binary variable (severe dengue (DHF/DSS) vs DF). In the adjusted model, the association between the hyperdestructive phenotype and bleeding was attenuated and no longer statistically significant (adjusted OR 2.83, 95% CI 0.59-13.60, p = 0.194). Similarly, the hypoproductive phenotype (adjusted OR 0.56, 95% CI 0.12-2.60, p = 0.460) and the lazy-suppressed phenotype (adjusted OR 2.58, 95% CI 0.33-20.33, p = 0.368) remained non-significant.

Among covariates, severe dengue emerged as the strongest independent predictor of bleeding (adjusted OR 41.83, 95% CI 14.41-121.46, p < 0.001), whereas age (p = 0.123), sex (p = 0.563), and platelet count (p = 0.916) were not significantly associated.

ROC analysis for bleeding prediction

**Figure 3:**
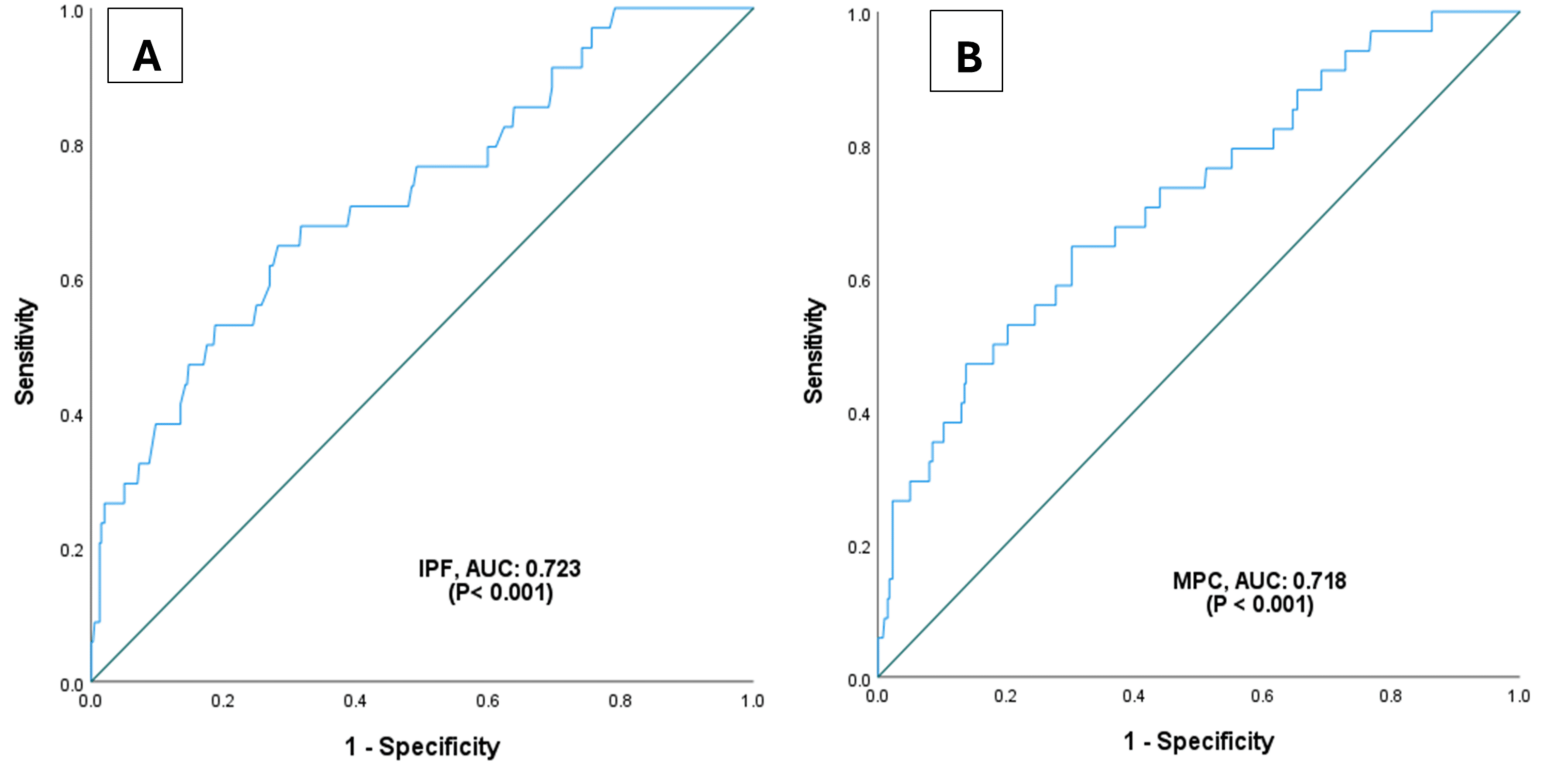
ROC curves demonstrating the diagnostic performance of platelet indices for predicting bleeding in dengue patients. (A) ROC for immature platelet fraction (IPF), showing moderate discriminatory ability with an AUC of 0.723 (p < 0.001). (B) ROC for mean platelet component (MPC), also demonstrating moderate diagnostic performance with an AUC of 0.718 (p < 0.001). These findings suggest that platelet kinetics–based indices provide clinically relevant information for bleeding risk stratification, although their standalone predictive accuracy remains moderate. ROC: receiver operating characteristic; AUC: area under the curve

ROC analysis was performed to evaluate the diagnostic performance of platelet indices in predicting bleeding in dengue patients. IPF showed good discriminatory ability, with an AUC of 0.723 (95% CI 0.631-0.815, p < 0.001) (Figure 3A), indicating moderate accuracy in distinguishing patients with and without bleeding. The optimal cutoff value, determined using the Youden index, was approximately 0.53%. At this threshold, IPF achieved a sensitivity of 97.1%, a specificity of 21.7%, and a Youden index of 0.188.

MPC showed good discriminatory ability, with an AUC of 0.718 (95% CI 0.625-0.810, p < 0.001) (Figure 3B), indicating moderate accuracy in distinguishing patients with and without bleeding. The optimal cutoff value, determined using the Youden index, was approximately 0.0495. At this threshold, MPC achieved a sensitivity of 97.1%, a specificity of 13.7%, and a Youden index of 0.108.

Dengue bleeding risk score

**Figure 4:**
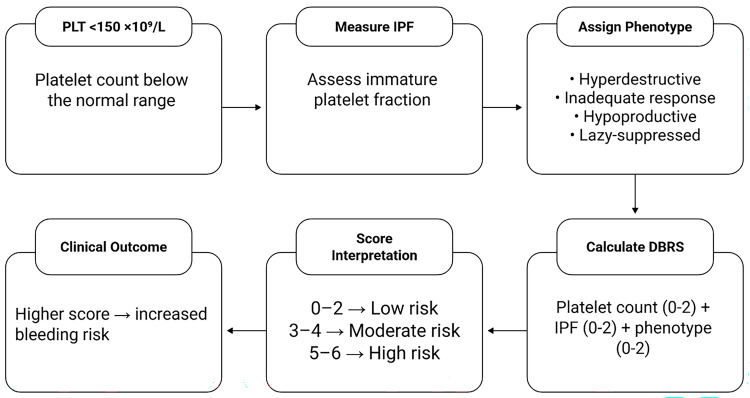
Diagnostic algorithm for bleeding risk stratification using platelet indices and thrombocytopenia phenotypes. The flowchart illustrates a stepwise approach for evaluating bleeding risk in dengue patients. Following confirmation of thrombocytopenia (PLT <150 ×10⁹/L), IPF is assessed to determine platelet kinetics. Patients with elevated IPF (>0.53%) are further evaluated for phenotype classification, while those with low IPF are categorized as hypoproductive or suppressed states. PLT, platelet count; IPF, immature platelet fraction; DBRS, Dengue Bleeding Risk Score Figure created by the authors using Microsoft PowerPoint (Microsoft Corporation, Redmond, Washington, United States).

To improve clinical risk sorting, a composite DBRS was developed to improve clinical risk classification by incorporating platelet indices and characteristics of thrombocytopenia. The scoring model integrated essential characteristics derived from the analysis, including IPF (>0.53%), MPC (>0.049), platelet count (<50 ×10⁹/L), and thrombocytopenia phenotype. Each parameter received a weighted score according to its correlation with bleeding: IPF >0.53% (+2), MPC >0.049 (+1), platelet count <50 ×10⁹/L (+2), hyperdestructive phenotype (+3), and inadequate response phenotype (+1), whereas hypoproductive and lazy-suppressed phenotypes were assigned a score of 0. The cumulative score ranged from 0 to ≥5, facilitating classification into three clinically significant categories: low risk (0-2 points), moderate risk (3-4 points), and severe bleeding risk (≥5 points). Significantly elevated DBRS values were primarily noted in patients with hyperdestructive thrombocytopenia and were closely linked to the incidence of hemorrhagic symptoms (Figure 4).

## Discussion

This study illustrates that platelet indices offer therapeutically significant insights into the etiology of thrombocytopenia in dengue and are closely correlated with bleeding risk. The results indicate that thrombocytopenia in dengue is not a uniform phenomenon but predominantly reflects multiple mechanistic phenotypes, including hyperdestructive, inadequate response, hypoproductive, and lazy-suppressed states, with the hyperdestructive phenotype becoming more prominent in severe disease [4-10]. This is corroborated by the gradual escalation of hyperdestructive thrombocytopenia from DF to DHF and DSS, in conjunction with the significant increase in IPF and the decrease in PLT and PCT noted in our study.

A significant discovery is the gradual transition to hyperdestructive thrombocytopenia correlated with escalating illness severity, challenging the notion that bone marrow suppression alone explains severity. This observation corroborates the idea that severe dengue is influenced by dysregulated immunological activation, endothelial dysfunction, and heightened platelet clearance, rather than solely by bone marrow suppression [4]. At the same time, the predominance of the inadequate response phenotype (55.7%) suggests that many patients exhibit a partial but insufficient compensatory thrombopoietic response, representing an intermediate biological state between destruction and impaired production.

The substantial increase of IPF in hyperdestructive thrombocytopenia underscores its function as an indicator of bone marrow compensation. This finding aligns with other research indicating that IPF signifies heightened thrombopoietic activity due to peripheral platelet breakdown [6,10]. Conversely, hypoproductive and lazy-suppressed phenotypes were characterized by low IPF levels, indicating impaired or minimal marrow response. These findings reinforce the concept that platelet kinetics, rather than absolute platelet count alone, better reflect disease activity and bleeding risk.

Importantly, in our study, bleeding risk differed significantly across thrombocytopenia phenotypes, with the hyperdestructive phenotype demonstrating the highest frequency of hemorrhagic manifestations. In the unadjusted analysis, this phenotype was strongly associated with bleeding. However, after adjustment for disease severity and other covariates, this association was attenuated and no longer statistically significant. This indicates that the relationship between hyperdestructive thrombocytopenia and bleeding is largely driven by its strong association with severe dengue, which emerged as the dominant independent predictor of bleeding. This finding has important clinical implications. It suggests that while platelet kinetics contribute to bleeding risk, disease severity remains the primary determinant, and thrombocytopenia phenotype should be interpreted within the broader clinical context. This also explains why platelet count alone is insufficient for predicting bleeding, as it does not capture the underlying pathophysiological processes driving disease severity.

Coagulation parameters exhibited minimal variations across thrombocytopenia phenotypes, suggesting that coagulation issues are not the principal contributors to bleeding in this context. This is aligned with existing research indicating that platelet dysfunction and vascular variables significantly contribute to dengue-related bleeding [4,11].

The present study also demonstrated that platelet indices, particularly IPF and MPC, reflect platelet turnover and functional status and showed moderate performance in predicting bleeding, rather than serving as independent discriminators of thrombocytopenia phenotypes. This indicates that while these indices are biologically informative, they are insufficient as standalone predictors and should be integrated with clinical and laboratory parameters. Increased IPF has consistently correlated with heightened platelet turnover in consumptive thrombocytopenia, serving as a reliable indicator for distinguishing hyperdestructive conditions from impaired production [5,12]. MPC indicates the internal composition and activation status of platelets rather than their formation kinetics, which may account for its marginally weaker, yet still substantial, discriminating capability. However, when applied to bleeding prediction, both IPF and MPC demonstrated only moderate discriminatory performance, highlighting that single biomarkers are insufficient for accurate clinical prediction. This supports the need for integrative approaches.

The simultaneous application of IPF and MPC offers a comprehensive assessment of thrombocytopenia by integrating both quantitative and qualitative platelet changes. Building on this, the DBRS proposed in this study integrates platelet indices, platelet count, and thrombocytopenia phenotype into a unified model, potentially providing a more comprehensive approach to risk stratification than platelet count alone. The DBRS should be considered a proposed mechanism-based risk stratification tool that requires prospective validation in independent cohorts before routine clinical application. Therefore, its clinical utility should be regarded as preliminary and interpreted cautiously until validated in external populations. This is particularly relevant in resource-limited settings, where simple and accessible evaluation tools may support clinical decision-making.

The significant correlation between hyperdestructive thrombocytopenia and bleeding underscores the inadequacy of relying solely on platelet count, as it does not accurately reflect the underlying pathophysiological mechanisms of thrombocytopenia. Dengue infection is characterized by complex mechanisms, including immune-mediated platelet destruction, bone marrow suppression, and endothelial dysfunction, all of which influence platelet dynamics and function [5,13]. The capacity of IPF and MPC to represent various processes underscores their function as integrative biomarkers. Moreover, analogous results have been reported in recent dengue research, indicating that platelet indices, such as MPV, PDW, and IPF, correlate with disease severity and hemorrhagic risk [14,15].

In areas such as the Red Sea State, where dengue is widespread and healthcare resources are limited, implementing routine platelet indicators could markedly improve clinical management. This method facilitates mechanistic diagnosis and risk assessment by providing accessible test data, thereby promoting more precise and effective care.

This study, however, possesses limitations. The application of IPF to characterize thrombocytopenia phenotypes may partially elucidate diagnostic performance, but the study-derived thresholds require external validation. In addition, the proposed DBRS was developed from a single cohort and was not internally or externally validated; therefore, its clinical utility should be considered preliminary. The limited number of DSS patients limits generalizability across severe illness classifications, and the small size of the hyperdestructive group may compromise the stability of regression estimates and increase the risk of model overfitting. Although multivariable analyses adjusted for age and sex, residual confounding cannot be completely excluded. Furthermore, the IPF classification thresholds and DBRS weighting scheme were study-derived and based on observed associations, diagnostic performance, and biological plausibility; therefore, they should be considered exploratory and require confirmation in larger independent cohorts.

## Conclusions

This study illustrates that thrombocytopenia in dengue is a heterogeneous, mechanism-driven phenomenon, rather than merely a fall in PLT. The recognition of four distinct phenotypes, hyperdestructive, inadequate response, hypoproductive, and lazy-suppressed, provides a framework for understanding platelet dynamics in dengue. The hyperdestructive phenotype was significantly linked to bleeding, regardless of platelet count, underscoring the essential significance of platelet turnover and functional changes in the development of hemorrhage. Conversely, hypoproductive and repressed phenotypes did not exhibit a significant correlation with heightened bleeding risk.

While platelet indicators, such as IPF and MPC, exhibited good discriminatory performance for thrombocytopenia phenotypes, their predictive value for bleeding was moderate, highlighting the need for comprehensive strategies. The suggested DBRS, which integrates platelet indices, PLT, and phenotypic classification, represents a preliminary mechanism-oriented approach to clinical risk stratification that requires validation in independent cohorts before routine clinical application. These findings support further investigation of mechanism-oriented approaches beyond PLT alone, particularly in resource-constrained environments. Given the observational nature of the study, the reported findings should be interpreted as associations rather than evidence of causality. Additional validation in large cohorts is necessary to verify the clinical efficacy of this approach.
